# Targeting pseudoknots with Cas13b inhibits porcine epidemic diarrhoea virus replication

**DOI:** 10.1099/jgv.0.002071

**Published:** 2025-02-04

**Authors:** Hee-Jeong Han, Daseuli Yu, Jeonghye Yu, Jihye Kim, Won Do Heo, Dongseob Tark, Sang-Min Kang

**Affiliations:** 1 Laboratory for Infectious Disease Prevention, Korea Zoonosis Research Institute, Jeonbuk National University, Iksan 54531, Republic of Korea; 2 ViEL-T Corporate Research Institute, ViEL-T lnc., Jeonju Innovation Startup Hub (SJ Bldg) 204, Jeonju 54852, Republic of Korea; 3 Department of Biological Sciences, Korea Advanced Institute of Science and Technology, Daejeon 34141, Republic of Korea

**Keywords:** CRISPR-Cas13b, CRISPR RNAs (crRNAs), porcine epidemic diarrhoea virus (PEDV), pseudoknot, RNA editing, viral replication

## Abstract

Clustered regularly interspaced short palindromic repeats-associated protein 13 (CRISPR-Cas13), an RNA editing technology, has shown potential in combating RNA viruses by degrading viral RNA within mammalian cells. In this study, we demonstrate the effective inhibition of porcine epidemic diarrhoea virus (PEDV) replication and spread using CRISPR-Cas13. We analysed the sequence similarity of the pseudoknot region between PEDV and severe acute respiratory syndrome coronavirus 2, both belonging to the *Coronaviridae* family, as well as the similarity of the RNA-dependent RNA polymerase (RdRp) gene region among three different strains of the PED virus. Based on this analysis, we synthesized three CRISPR RNAs (crRNAs) targeting the pseudoknot region and the nonpseudoknot region, each for comparison. In cells treated with crRNA #3 targeting the pseudoknot region, *RdRp* gene expression decreased by 95%, membrane (*M*) gene expression by 89% and infectious PEDV titre within the cells reduced by over 95%. Additionally, PED viral nucleocapsid (*N*) and M protein expression levels decreased by 83 and 98%, respectively. The optimal concentration for high antiviral efficacy without cytotoxicity was determined. Treating cells with 1.5 µg of Cas13b mRNA and 0.5 µg of crRNA resulted in no cytotoxicity while achieving over 95% inhibition of PEDV replication. The Cas13b mRNA therapeutics approach was validated as significantly more effective through a comparative study with merafloxacin, a drug targeting the pseudoknot region of the viral genome. Our results indicate that the pseudoknot region plays a crucial role in the degradation of the PEDV genome through the CRISPR-Cas13 system. Therefore, targeting Cas13b to the pseudoknot offers a promising new approach for treating coronavirus infections.

## Data Availability

We have provided the original uncropped and unadjusted image and blot results reported in an ‘original figure raw image’ file, available in the online Supplementary Material.

## Introduction

Clustered regularly interspaced short palindromic repeats (CRISPR) and CRISPR-associated protein 13 (Cas13) can provide microbial defence against foreign RNAs through RNA-induced endonuclease activity [1,5]. This technology represents a novel platform for RNA editing, especially in combating diverse RNA viruses. Recent advancements have expanded the system’s capability to recognize and degrade viral RNA within mammalian cells, markedly enhancing its research and application potential [6,7].

Veterinary viral diseases have had substantial impacts on animal health, economic stability and public health. For example, South Korea reported 1131 cases of porcine epidemic diarrhoea virus (PEDV), with periodic outbreaks occurring from winter to spring in 2013–2014, 2017–2018, 2018–2019 and 2021–2022, and the most affected regions being Chungnam, Jeonbuk and Jeju. Suckling piglets are especially susceptible to PEDV, with infection causing severe diarrhoea, dehydration and high mortality rates, profoundly affecting the swine industry due to major losses [8].

PEDV classified under the *Alphacoronavirus* genus within the *Coronaviridae* subfamily of the *Coronaviridae* family shares structural features with severe acute respiratory syndrome coronavirus 2 (SARS-CoV-2), despite the viruses belonging to different genera within the same order, family and subfamily [9]. Notably, both viruses feature a pseudoknot structure in the *ORF1b* gene that plays a pivotal role in their replication and pathogenicity.

The conservation of RNA structures among these viruses is crucial for understanding their molecular mechanisms. All coronaviruses employ programmed −1 ribosomal frameshift (−PRF) to regulate protein expression. Studies on SARS-CoV-2 have identified a distinctive three-stemmed mRNA pseudoknot that induces high −PRF rates [10]. The −PRF process is crucial for synthesizing viral RNA-dependent RNA polymerase (RdRp or Nsp12) and other nonstructural proteins involved in viral RNA capping, modification, processing and proofreading [11]. Thus, targeting −PRF activity presents a potential therapeutic strategy to impair viral replication [12]. Previous research has shown that Cas13b mRNAs and CRISPR RNAs (crRNAs) targeting the pseudoknot region effectively inhibit SARS-CoV-2 replication [13].

This study investigated whether targeting the PEDV pseudoknot region using the CRISPR-Cas13 system effectively inhibits viral spread. Synthetic spacers complementary to both the pseudoknot and nonpseudoknot sequences of PEDV were used. The findings of this study suggest that targeting the pseudoknot regions is more effective in inhibiting PEDV transmission compared with targeting the nonpseudoknot regions.

## Methods

### Cell lines and culture conditions

Vero cells were cultured in Dulbecco’s modified Eagle’s medium (DMEM) supplemented with high glucose, pyruvate, 10% foetal bovine serum (FBS), 100 U ml^−1^ penicillin and 100 U ml^−1^ streptomycin. Cells were cultured at 37 °C in a 5% CO_2_ incubator.

### PEDV infection and titration

An outbreak of PEDV was reported in Pocheon, Gyeonggi-do, from where intestinal samples were collected from the infected pigs. The PEDV strain was isolated from the collected porcine intestinal tissues and named CKK1-1 (PEDV; GenBank: OM714830.2) [14]. This procedure was conducted at the Jeonbuk National University Veterinary Diagnostic Center. For PEDV production, Vero cells were infected with PEDV (CKK1-1) and cultured in DMEM without FBS and antibiotics at 37 °C in a 5% CO_2_ incubator. Upon observation of cytopathic effects, virus-containing supernatants were clarified via centrifuged at 3000 r.p.m. for 10 min, removing all cell debris. Viral titres were determined through a tissue culture infective dose 50 (TCID_50_) assay using the Reed–Muench method.

### Design and synthesis of crRNAs

To design efficient crRNAs, the crRNA targeting prediction programs CHOPCHOP (https://chopchop.cbu.uib.no) and Cas13design (https://cas13design.nygenome.org/) were employed. crRNAs targeting PEDV were designed using these programs, with three crRNAs selected for both the pseudoknot region and the nonpseudoknot region. Designed crRNAs were synthesized by Integrated DNA Technologies with 2′-O-methyl-3′-phosphorothioate modifications at both the 5′ and 3′ ends of the three terminal nucleotides. The sequences of the crRNAs designed to target PEDV are summarized in Table 1.

**Table 1.** Sequences of crRNAs designed to target PEDV

No.	crRNA name	Targeting region	Sequence
1	**PEDV crRNA#1**	RdRp_Pseudoknot	CGA GCU GCA CUA GAG CCC CGU ACU CGU UUA
2	**PEDV crRNA#2**	RdRp_Pseudoknot	AUC AGU ACC AUU ACA GGG CUC UAG UCG AGC
3	**PEDV crRNA#3**	RdRp_Pseudoknot	GGA UUG CUC GUG UUC CAU CGC AGA CUU GGU
4	**PEDV crRNA#4**	RdRp_non-Pseudoknot	GCA CUA UUU GAU ACA CGA CGC GAG CUU GGC
5	**PEDV crRNA#5**	RdRp_non-Pseudoknot	CGA CAA UCU GCA UAG UAU GCU GCG AGC AAA
6	**PEDV crRNA#6**	RdRp_non-Pseudoknot	CAG AAA AAG ACU CUA ACA CAC CAG CAU UCA

### mRNA and crRNA transfection

Vero cells were seeded at 2×10^5^ cells per well in 12-well plates for 16 h. Before transfection, the culture medium was replaced with a fresh medium. Transfection was then performed using jetMESSENGER (Polyplus) with 0.5, 1, 1.5 and 2 µg of Cas13b mRNA and 0.1, 0.2, 0.5 and 1 µg of crRNA per well, respectively.

### Quantitative reverse-transcription PCR

For RNA analysis, total RNA was isolated using TRIzol reagent (Invitrogen, Carlsbad, CA), and its concentration was determined using NanoDrop One/OneC. Equal amounts of RNA were quantified and reverse-transcribed into cDNA using CellScript cDNA Master Mix/Premix (CellSafe, Gyeonggi-do, South Korea). Quantitative reverse-transcription PCR (qRT-PCR) was performed using 1 µl of cDNA, iQ SYBR Green Supermix (Bio-Rad) and 5 pmol of gene-specific primers. PCR cycling conditions included 40 cycles of 95 °C for 20 s and 58 °C for 40 s. The primers used targeted the PEDV membrane (M) gene region (forward primer: 5′-GGTTCTATTCCCGTTGATGAGGT-3′; reverse primer: 5′-AACACAAGAGGCCAAAGTATCCAT-3′) and the RdRp (nsp12) region (forward primer: 5′- TTCACTTGGAAGGATGGTCGTG-3′; reverse primer: 5′- CTCCTCACAAGCACCTACCTTAA-3′). The SARS-CoV-2 gene was normalized to *β*-actin (forward primer: 5′-TGACAGCAGTCGGTTGGAGCG-3′; reverse primer: 5′-GACTTCCTGTAACAACGCATCTCATA-3′). Relative gene expression was calculated using the 2-ΔΔCt method, with the untreated control employed as the reference.

### Immunofluorescence assay

Vero cells were washed using PBS, fixed using methanol and acetone (1 : 1 ratio) for 10 min, washed three times with PBS and blocked with 5% bovine serum albumin for 1 h. Cells were then incubated with anti-PEDV monoclonal antibody (Cat. No. 9191, Median Diagnostics, South Korea) for 2 h, followed by incubation with the secondary antibody (#4488, anti-mouse IgG fragment conjugated with Alexa Fluor 488, Cell Signaling Technology). Nuclei were stained with DAPI (no. 4083S; Cell Signaling Technology), and fluorescent images were captured using the CELENA X digital imaging system (Logos Biosystems). Stained cells were quantified using the Indica Labs FISH-IF module.

### Immunoblot assay

To detect PEDV proteins, PEDV-infected cells were washed with cold PBS and lysed with cold RIPA lysis buffer [25 mM Tris-HCl (pH 7.6), 150 mM sodium chloride, 1% Nonidet P-40, 1% sodium deoxycholate and 0.1% SDS; Thermo Fisher Scientific], supplemented with a protease and phosphatase inhibitor cocktail (Thermo Fisher Scientific). The lysates were then centrifuged at 13 000 r.p.m. and 4 °C for 15 min. Protein samples were mixed with 4× Laemmli sample buffer containing 2-mercaptoethanol and subsequently heated at 98 °C for 10 min. Proteins were separated via SDS-polyacrylamide gel electrophoresis and electrotransferred to a polyvinylidene difluoride membrane. The membrane was blocked in PBS containing 5% BSA for 1 h at room temperature. Subsequently, it was incubated with anti-PEDV antibody (MBS560870; MyBiosource) and GAPDH antibody (sc-47724; Santa Cruz Biotechnology) for 2 h at room temperature on a shaker. After incubation, the membrane was washed with 0.1% Tris-buffered saline/Tween and incubated with anti-mouse IgG-HRP (Cell Signaling Technology). Proteins were detected using an ECL kit (ELPIS Biotech).

### Plaque assay

Plaque assay was performed as previously described [13]. Briefly, Vero cells were seeded into 12-well plates at 3×10^5^ cells per well and incubated at 37 °C for 16 h in a 5% CO_2_ incubator. The virus-containing supernatant was diluted tenfold in DMEM without FBS and antibiotics. Cells were infected with tenfold serial dilutions of the virus (1 ml) for 1 h. After washing with DMEM without FBS and antibiotics, the cells were overlaid with a growth medium containing 0.6% low-melting agarose. After 96 h, the cells were fixed with 3.65% formaldehyde at 37 °C for 1 h in 5% CO_2_ and subsequently washed with tap water to remove formaldehyde and agarose. Finally, the cells were stained with 0.5% crystal violet, and the plaques were counted and quantified.

### Cell cytotoxicity assay

Vero cells were seeded into 96-well plates at 2×10^4^ cells per well and incubated for 16 h at 37 °C in a 5% CO_2_ incubator. Cells were transfected with Cas13b mRNA and crRNAs using jetMESSENGER (Polyplus). Additionally, 10 mM merafloxacin dissolved in dimethylsulfoxide was diluted to 20, 50 and 100 µM in DMEM containing 10% FBS, after which 100 µl of merafloxacin was added to the cells. After 48 h, cell viability was assessed by measuring ATP levels using the CellTiter-Glo 2.0 Cell Viability Assay Kit (Promega).

### Effect of merafloxacin on PEDV-infected cells

Vero cells were plated at 2×10^5^ cells per well in a 12-well plate 24 h before infection. Vero cells were infected with PEDV at an m.o.i. of 0.05 for 2 h at 37 °C. Merafloxacin was diluted to 20, 50 and 100 µM using DMEM containing 10% FBS. The diluted merafloxacin was added to each of the infected cells. For PEDV-infected Vero cells, the viral RNA and protein were quantified using qRT-PCR and IFA, respectively, after 24 h.

## Results

### Design of crRNA targeting pseudoknot or nonpseudoknot for PEDV

**Fig. 1.**
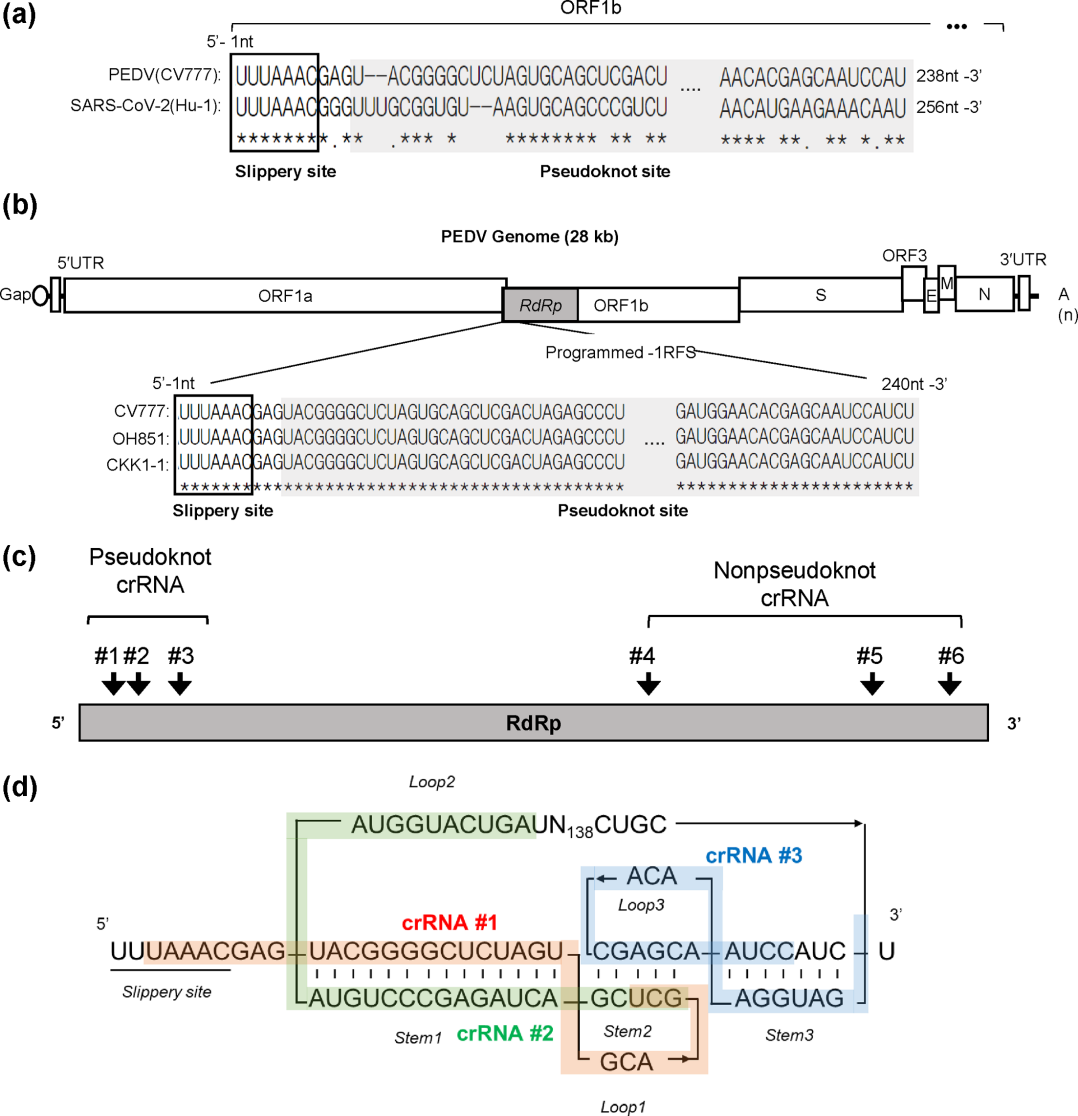
Comparison of PEDV variant pseudoknot sequences and crRNA production. (**a**) Alignment of slippery and pseudoknot sequences between SARS-CoV-2 and PEDV. The alignment compares nucleotide sequences, highlighting the slippery site (boxed) and pseudoknot site (grey) between SARS-CoV-2 and PEDV. Identical nucleotides are marked with asterisks (*) and differences with periods (.) below the sequences. Gaps are shown as dashes (-). (**b**) Sequence diversity among PEDV variants, including CV777, OH851 and CKK1-1. The top diagram shows the general genome organization of PEDV. The bottom diagram shows the multiple sequence alignment generated via CLUSTALW that compares the slippery region (boxed) and pseudoknot region (grey) among three PEDV variants. (**c**) CRISPR-Cas13b system targeting the *RdRp*) gene of PEDV. The diagram illustrates CRISPR-Cas13b targeting the *RdRp* gene, indicating the pseudoknot and nonpseudoknot crRNA sites. (**d**) Schematic of the secondary structures of the PEDV slippery and pseudoknot regions with crRNA targeting sites marked. Sequences targeted by crRNAs #1, #2 and #3 are indicated by the red, green and blue bands, respectively.

Although few studies have investigated the nucleotide sequences and function of the PEDV pseudoknot structure compared with those of SARS-CoV-2, this structure plays a similar role to that of SARS-CoV-2. Sequence alignment of PEDV strain CV777 and SARS-CoV-2 strain Hu-1 revealed complete conservation of the slippery region sequence UUUAAAC between the two viruses. However, the notable similarity was not observed in the pseudoknot site, which is crucial for the −PRF process (Fig. 1a). The RdRp genes of the two viruses exhibit a relatively low identity of ~60.01%. For the design of crRNAs targeting the CRISPR-Cas13 system, a multiple sequence alignment was performed on the slippery sequence and pseudoknot sequence of RdRp from PEDV strains including CV777 (earliest identified strain), OH851 (a highly virulent strain prevalent across Asia) and CKK-1 [14] (isolated in Korea and is closely similar to O851). The findings revealed that the slippery sequences and pseudoknot sequences are highly conserved, except for two nucleotides among the three PEDV strains (Fig. 1b). Two crRNA prediction programs (CHOPCHOP and Cas13design) were employed to design crRNA targeting 2780 nucleotides of PEDV *RdRp* [15,17]. A total of six crRNAs were designed, including three targeting the non-pseudoknot region (crRNA #4–6) and three targeting the pseudoknot region (crRNA #1–3) (Fig. 1c). The crRNA#1 targets stem1, and crRNA#2 targets stem2 of the conserved pseudoknot region, whereas crRNA#3 targets both stem2 and stem3 (Fig. 1d, Table 1).

### Cas13b effectively blocks PEDV propagation upon pseudoknot-targeting in *ORF1b*

**Fig. 2.**
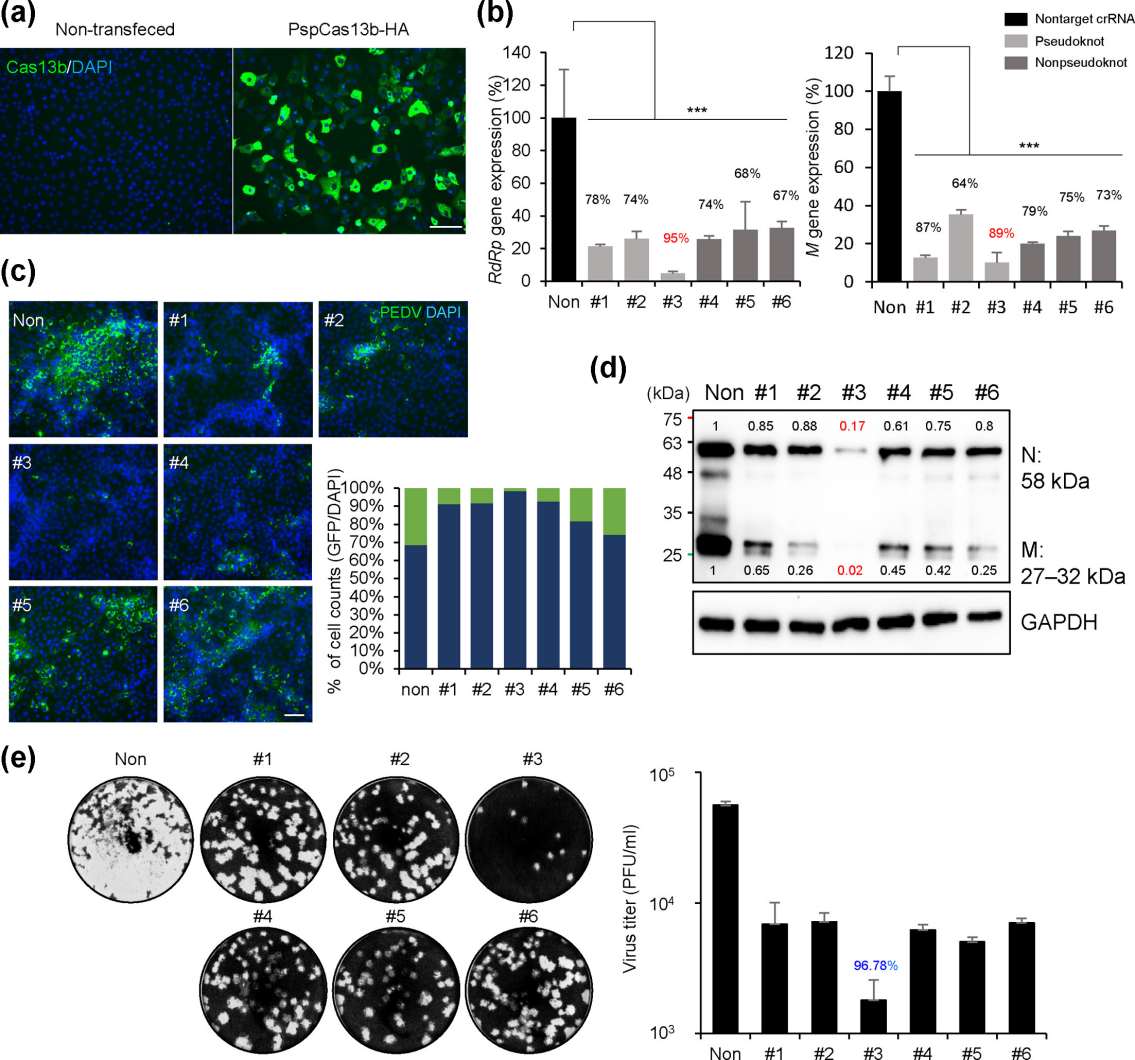
Potent inhibition of PEDV transmission via pseudoknot-targeting crRNA #3. (**a**) Quantification of PEDV gene expression. Vero cells were transfected with Cas13b mRNA and crRNAs targeting CKK1-1 of the PEDV strain 24 h before infection. After 24 h, RNA was extracted, and PEDV *RdRp* and membrane gene expression levels were quantified using qRT-PCR. Results were normalized to RNA expression in cells transfected with nontargeting crRNAs. (**b**) Representative images of PEDV protein (green) and DAPI (blue) staining in Vero cells. Viral protein levels from (**a**) were visualized under a fluorescence microscope. Scale bar: 50 µm. (**c**) Quantification of the percentage of PEDV protein-positive cells relative to DAPI-positive cells, tested as in (**b**). Green bars represent PEDV protein-positive cells compared with total cells (DAPI-positive cells). (**d**) Total proteins from the infected cells in (**a**) were analysed using a western blot assay. Intensity ratios of PEDV proteins, normalized to GAPDH, are shown above and below each corresponding protein band. (**e**) Quantification of PEDV titre. Vero cells were infected with the virus from (**a**). Titration was performed using a plaque assay at 4 dpi. The dilution factor multiplied by the plaque count was used to determine the virus titre (*n*=3 for each group). Values are means±sem. One-way ANOVA was used for statistical analysis. ***P<0.0001.

To assess the efficacy of the Cas13b system targeting the pseudoknot structure of PEDV *ORF1b*, Cas13b mRNA and the designed crRNAs were transfected into Vero cells, followed by PEDV infection (m.o.i.=0.05). qRT-PCR analysis revealed that all six PEDV-targeting crRNAs suppressed the expression of both the *RdRp* and membrane-coding genes of PEDV. Notably, crRNA#3 reduced *RdRp* gene expression by 95% and *M* gene expression by 89% (Fig. 2a). The immunofluorescence assay showed that PEDV propagation efficacy decreased by >90% in cells treated with crRNAs #1–4, with crRNA #3 exhibiting the most potent inhibitory effect on PEDV (Fig. 2b, c). Immunoblot analysis with polyclonal antibody detecting PEDV proteins revealed an overall reduction in PEDV protein expression in cells treated with Cas13b mRNA and crRNAs. Specifically, crRNA #3 reduced N and M protein levels by 83 and 98%, respectively (Fig. 2d). A plaque assay confirmed that PEDV infectivity was reduced by 96.78% after treatment with crRNA #3. The crRNAs #1 and #2 targeting the pseudoknot region and crRNAs #4–6 targeting the nonpseudoknot region reduced PEDV titre by ~85% (Fig. 2e).

**Fig. 3.**
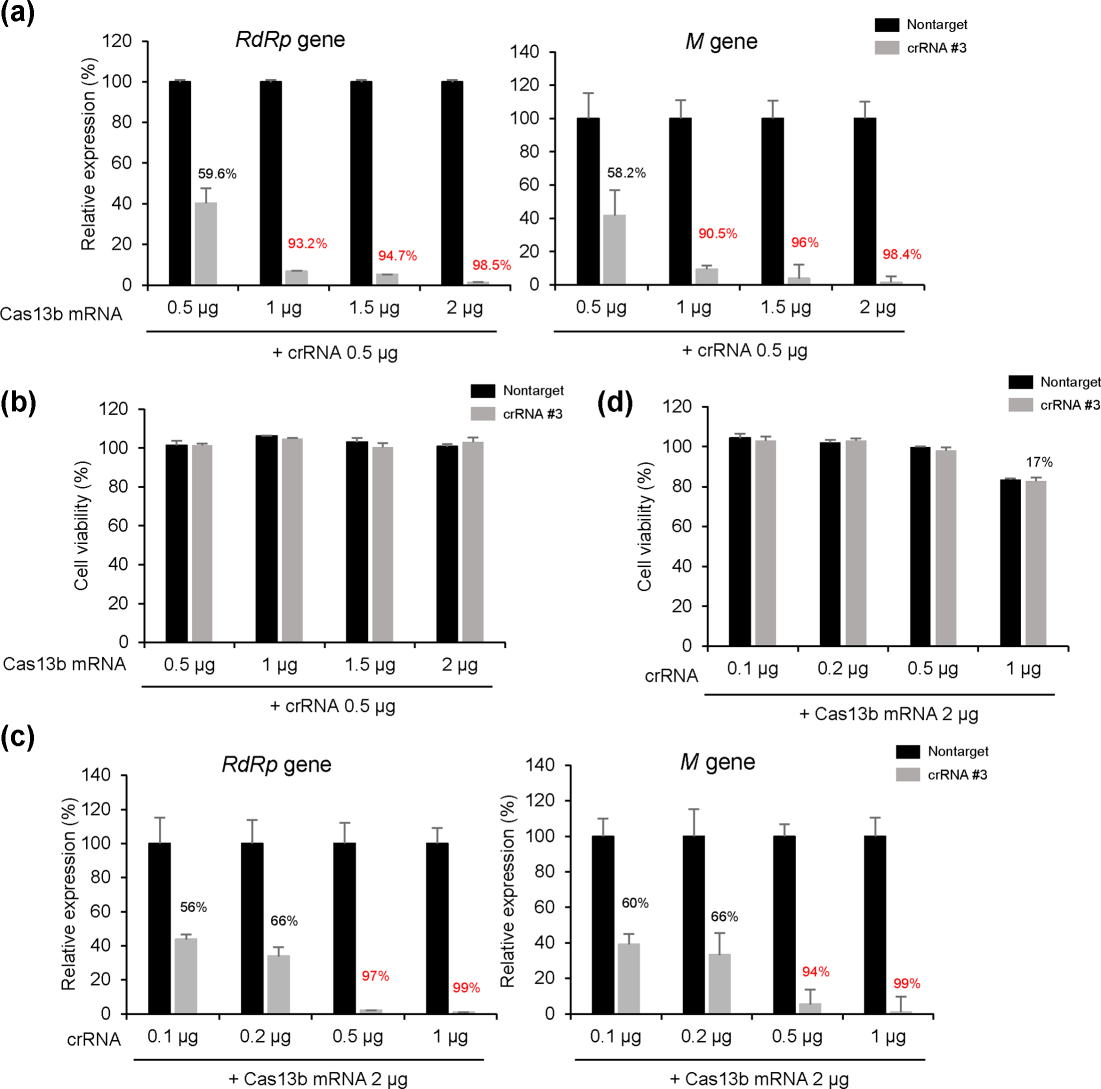
Optimization of Cas13b and crRNA for enhanced antiviral activity against PEDV in a dose-dependent manner. (a) Quantification of PEDV gene copies. Vero cells were transfected with 0.5 µg of either crRNA #3 or nontarget crRNA and varying amounts of Cas13b mRNA (0.5, 1.0, 1.5 or 2.0 µg). At 24 h post-transfection, cells were infected with PEDV (0.05 m.o.i.). After another 24 h, PEDV RdRp and membrane gene expression levels in total RNA were quantified using qRT-PCR. Results were normalized to RNA expression in cells transfected with nontargeting crRNAs. (b) The same RNA transfection from (a) was followed by a luminescent cell viability assay (ATP assay) 48 h later. Bioluminescence values were normalized to those of untreated cells. (c) Vero cells were transfected with 2 µg of Cas13b mRNA and various amounts of crRNA #3 (0.1, 0.2, 0.5 or 1.0 µg). After 24 h, the cells were infected with PEDV, and 24 h later, PEDV RdRp and membrane gene expression levels were quantified using qRT-PCR. Results were normalized to RNA expression in cells transfected with nontargeting crRNAs. (d) Luminescent cell viability assay (ATP assay) was performed 48 h after transfection, as shown in (c). Bioluminescence values were normalized to those of untreated cells. Values are means±sem. One-way ANOVA was used for statistical analysis. ***P<0.0001.

The optimal treatment concentration was determined by observing changes in PEDV RNA replication and cell viability at different concentrations of Cas13b mRNA and crRNAs. The expression of the PEDV *RdRp*- and membrane-coding genes gradually decreased with increasing Cas13b mRNA concentration under the 0.5 µg of crRNA #3. Cas13b mRNA (2.0 µg) and crRNA #3 (0.5 μg) showed no cytotoxicity and reduced viral gene expression by up to 98% (Fig. 3a, b). Next, the increasing crRNA #3 concentration under the 2 µg of Cas13b mRNA, RdRp and *M* genes decreased by 99% in cells transfected with 1 µg of crRNA #3 and 2 µg of Cas13b mRNA (Fig. 3c). However, a 17% reduction in cell viability indicated that cytotoxicity began to occur at the same concentration (Fig. 3d). These findings suggest that the Cas13b system effectively defends against PEDV by targeting the pseudoknot in PEDV *ORF1b*. Moreover, we determined the optimal concentration that inhibited more than 95% of PEDV replication without causing cytotoxicity.

### Merafloxacin, a pseudoknot formation inhibitor, simultaneously suppresses PEDV replication and induces cytotoxicity

**Fig. 4.**
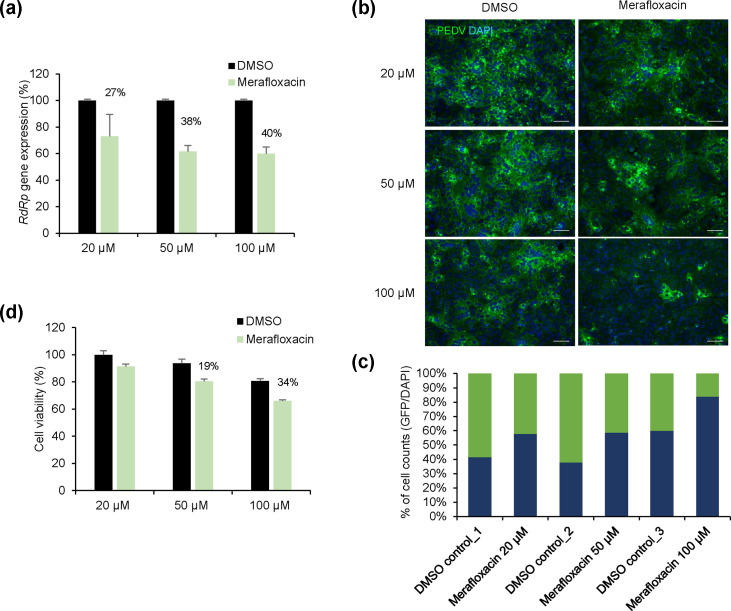
Inhibition of PEDV transmission via merafloxacin, a pseudoknot formation inhibitor. (**a**) Quantification of PEDV gene copies. Vero cells were infected with PEDV and treated with the indicated merafloxacin concentrations 1 h later. After 24 h, PEDV *RdRp* gene expression was quantified using qRT-PCR. Results were normalized to RNA expression in cells treated with DMSO. (**b**) Representative immunofluorescence images of PEDV protein (green) and DAPI (blue) staining in Vero cells. Images are from one of three replicates. Scale bars: 50 µm. (**c**) Quantification of the percentage of PEDV protein-positive cells relative to DAPI-positive cells, tested as in (**b**). (**d**) Cytotoxicity quantification following merafloxacin treatment. Vero cells were treated with the indicated concentrations of merafloxacin dissolved in DMSO. After 24 h, a luminescent cell viability assay (ATP assay) was performed. Bioluminescence values were normalized to those of untreated cells. Values are means±sem.

Merafloxacin, a fluoroquinolone antibacterial agent, inhibits pseudoknot formation, essential for the ribosomal frameshift in the *Betacoronavirus* genome. Previous studies demonstrated merafloxacin’s inhibition of SARS-CoV-2 propagation [11]; however, its effect on PEDV, an *Alphacoronavirus*, has not been reported. This study was the first to investigate whether merafloxacin inhibits viral propagation. These results showed ~40% reduction in PEDV RNA expression with 100 µM merafloxacin (Fig. 4a). Additionally, immunofluorescence assay confirmed that PEDV replication in cells decreased as the concentration of merafloxacin increased (Fig. 4b, c). However, cell viability also decreased by 34% with 100 µM merafloxacin (Fig. 4d). Therefore, merafloxacin exhibited lower antiviral efficacy against PEDV compared with Cas13b targeting the pseudoknot region.

## Discussion

Previous studies have established the CRISPR-Cas13b system’s potential as an effective antiviral therapy against SARS-CoV-2 by targeting its pseudoknot region within the *ORF1b* gene [13]. This approach not only confirmed the system’s effectiveness against SARS-CoV-2 but also extended its applicability to other coronaviruses. These results emphasize the versatility and broad-spectrum potential of this targeted antiviral strategy, especially in managing diverse coronavirus infections.

A prior study underscored the importance of designing crRNAs targeting single-stranded RNA regions for efficient RNA knockdown by Cas13, revealing that Cas13 targeting the pseudoknot structure in the long noncoding RNA (lncRNA) XIST transcript does not cleave RNA [18]. In the present study, crRNAs #1–3 were designed to target the PEDV pseudoknot region. Among these crRNAs, crRNA #3, which targets the complex H–H-type (kissing loop) structure involving interactions between short Loop1 of stem2 and long Loop3 of stem3, exhibited the most effective inhibition of PEDV propagation. Despite unclear underlying mechanisms, this study suggests that intermolecular forces between crRNA spacers and protospacers may outweigh base-pairing interactions in complex RNA structures, such as the H–H-type pseudoknot [19]. These findings suggest that viral inhibition efficacy can be improved by predicting RNA secondary and tertiary structures and designing crRNAs accordingly.

Merafloxacin, a fluoroquinolone, inhibits pseudoknot formation, which is crucial for frameshifting in betacoronaviruses [11]. However, its therapeutic efficacy against PEDV, an Alphacoronavirus, is limited, highlighting a fundamental limitation of chemical antivirals. Although human immunodeficiency virus 1 (HIV-1) pseudoknots play vital roles in viral replication and gene expression as well as the −PRF process [20], one study has shown that merafloxacin does not inhibit −PRF of HIV or viral replication, highlighting its narrow effectiveness against viruses with similar pseudoknot structures [21]. This limitation underscores the need for more flexible antiviral strategies, where the CRISPR-Cas13b system targeting pseudoknots shows substantial promise in reducing viral propagation across RNA viruses, offering a versatile alternative to traditional chemical antivirals.

Given that the CRISPR-Cas13 system is now widely recognized as an RNA interference technology, computational models have been developed to optimize crRNA selection. These screening models enable predictions of crRNA efficiency against target genes while minimizing off-target effects [15,17]. Despite these advancements, designing crRNAs for lengthy viral genomes remains challenging. The present study simplified crRNA design by focusing on the presence or absence of pseudoknot regions, achieving efficiency comparable to strategies considering high conservation scores, coding regions and the number of unpaired bases in RNA secondary structures [7,18, 22]. These findings highlight the potential of targeting pseudoknot regions as a straightforward yet potent antiviral therapy strategy.

All experiments in this study were conducted *in vitro*, necessitating future *in vivo* studies using piglets as experimental animals for PEDV research. Despite encountering various challenges, such as the expense of animal experiments and RNA synthesis as well as the efficiency of RNA delivery systems [23], *in vivo* research remains invaluable. To sustain high Cas13b expression levels, advanced technologies, such as self-amplifying mRNA (saRNA) and SINE-derived UP-regulation (SINEUP), have been proposed. saRNA incorporates additional RNA sequences enabling cellular replication, increasing mRNA levels and protein production over an extended period [24,25]. SINEUP, an lncRNA, enhances target mRNA translation efficiency [26]. Specifically, it binds to certain mRNAs, increasing their translational efficiency and boosting protein production. These technologies promise sustained therapeutic effects or robust gene expression for treatments requiring long-term efficacy.

In conclusion, targeting RNA virus pseudoknot regions with the CRISPR-Cas13b system presents a promising avenue for antiviral strategies. This study is the first to demonstrate effective PEDV propagation reduction *in vitro* using CRISPR-Cas13b, highlighting its potential as a novel approach for controlling viral infections.

## supplementary material

10.1099/jgv.0.002071 Supplementary Material 1.

## Abbreviations

CRISPR-Cas13: clustered regularly interspaced short palindromic repeats-associated protein 13
CRISPRs: clustered regularly interspaced short palindromic repeats
crRNA: CRISPR RNA
DMEM: Dulbecco’s modified Eagle’s medium
FBS: foetal bovine serum
HIV-1: human immunodeficiency virus 1
N: nucleocapsid protein
nsp: nonstructural proteins
PEDV: porcine epidemic diarrhoea virus
PRF: programmed ribosomal frameshift
qRT-PCR: quantitative reverse-transcription PCR
RdRp: RNA-dependent RNA polymerase
S: spike protein
saRNA: self-amplifying mRNA
SARS-CoV-2: severe acute respiratory syndrome coronavirus 2
SINEUP: SINE-derived UP-regulation
