# Supplementary figures and images for: Targeting pseudoknots with Cas13b inhibits porcine epidemic diarrhoea virus replication
Source: J Gen Virol. 2025 Feb 4;106(2):002071. doi: 10.1099/jgv.0.002071 (PMC11793167; doi:10.1099/jgv.0.002071)

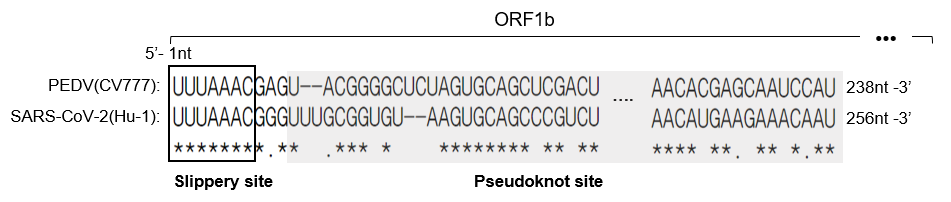

Supplement: Supplementary Material 1. [file jgv-106-02071-s001.zip › Fig 1/Fig 1 A.png]

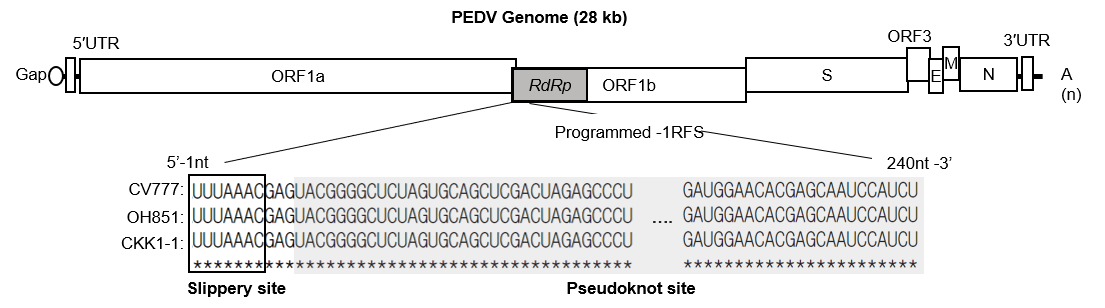

Supplement: Supplementary Material 1. [file jgv-106-02071-s001.zip › Fig 1/Fig 1 B.png]

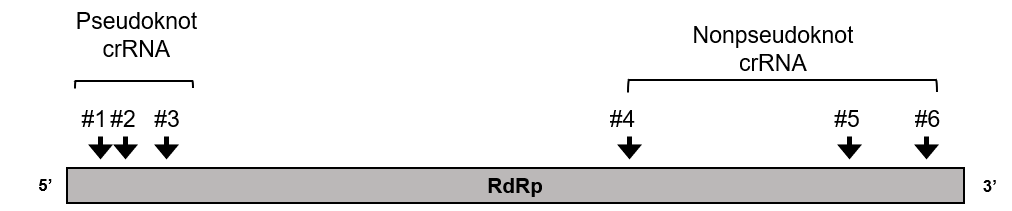

Supplement: Supplementary Material 1. [file jgv-106-02071-s001.zip › Fig 1/Fig 1 C.png]

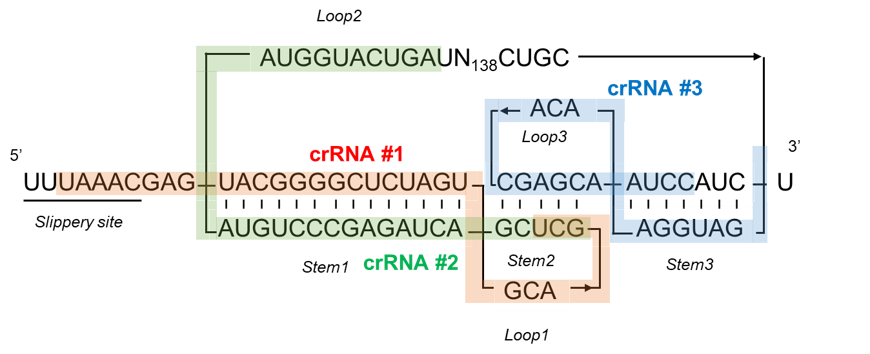

Supplement: Supplementary Material 1. [file jgv-106-02071-s001.zip › Fig 1/Fig 1 D.png]

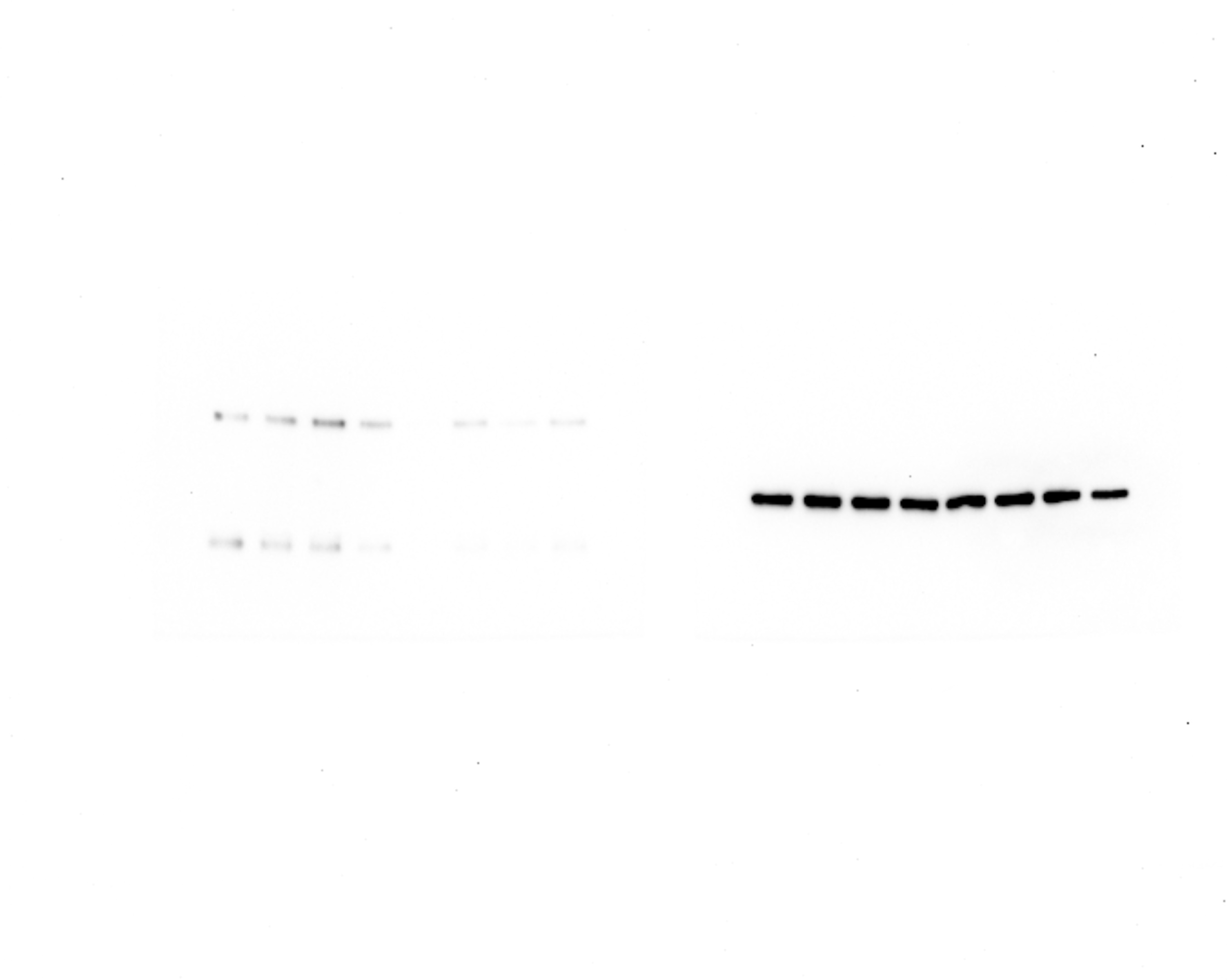

Supplement: Supplementary Material 1. [file jgv-106-02071-s001.zip › Fig 2/Fig 2 D/GAPDH_Chemi.jpg]

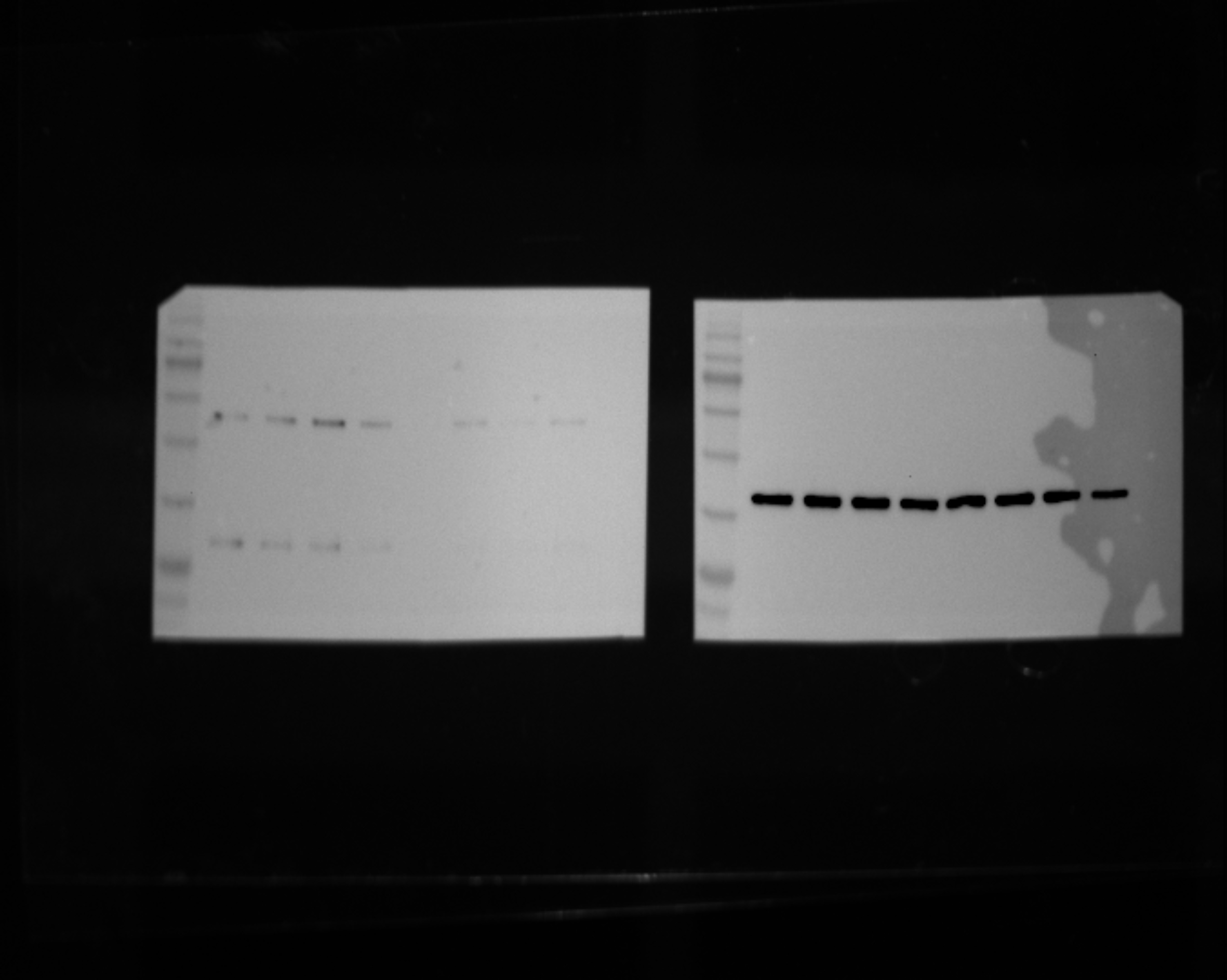

Supplement: Supplementary Material 1. [file jgv-106-02071-s001.zip › Fig 2/Fig 2 D/GAPDH_composite.jpg]

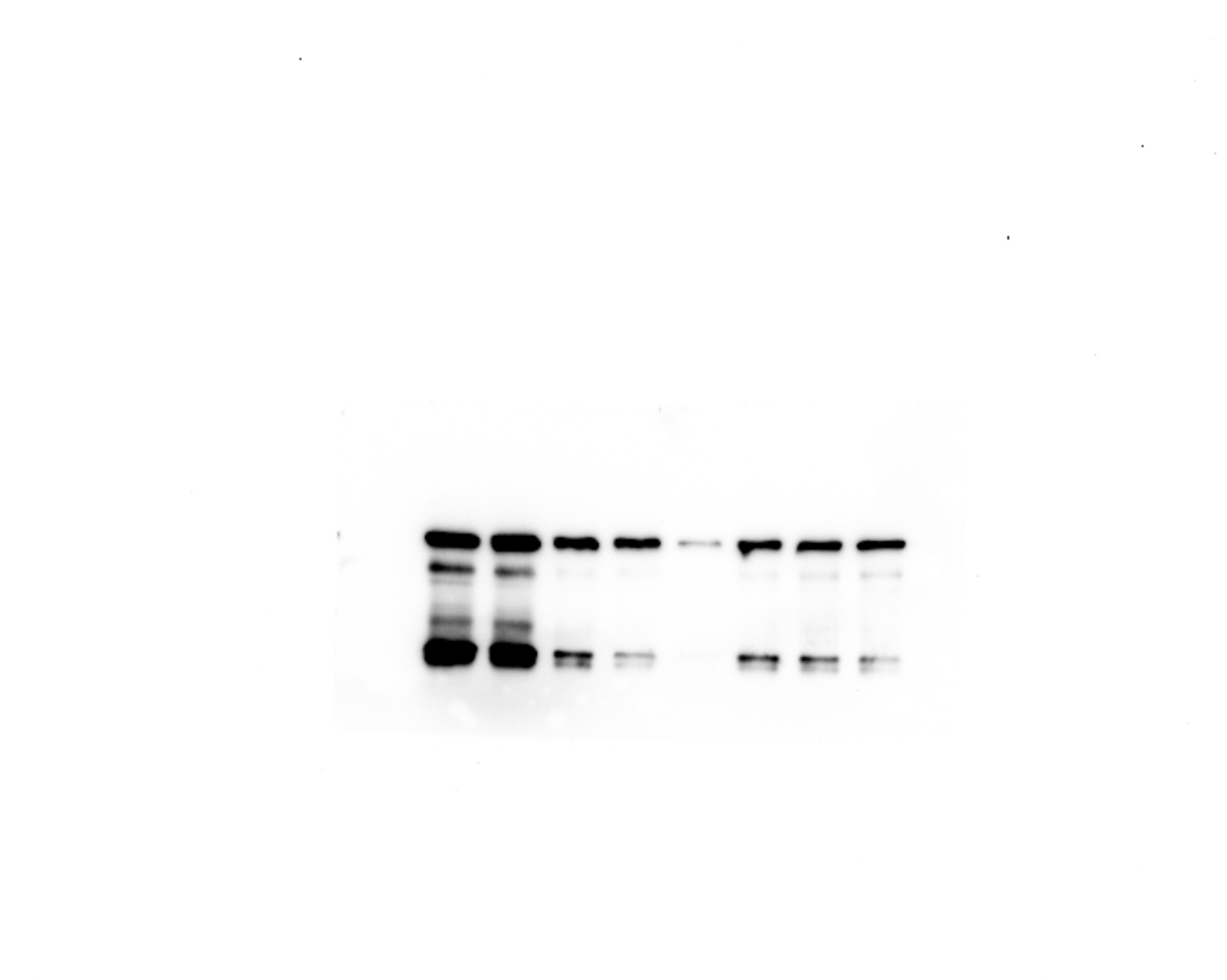

Supplement: Supplementary Material 1. [file jgv-106-02071-s001.zip › Fig 2/Fig 2 D/PEDV_Chemi.jpg]

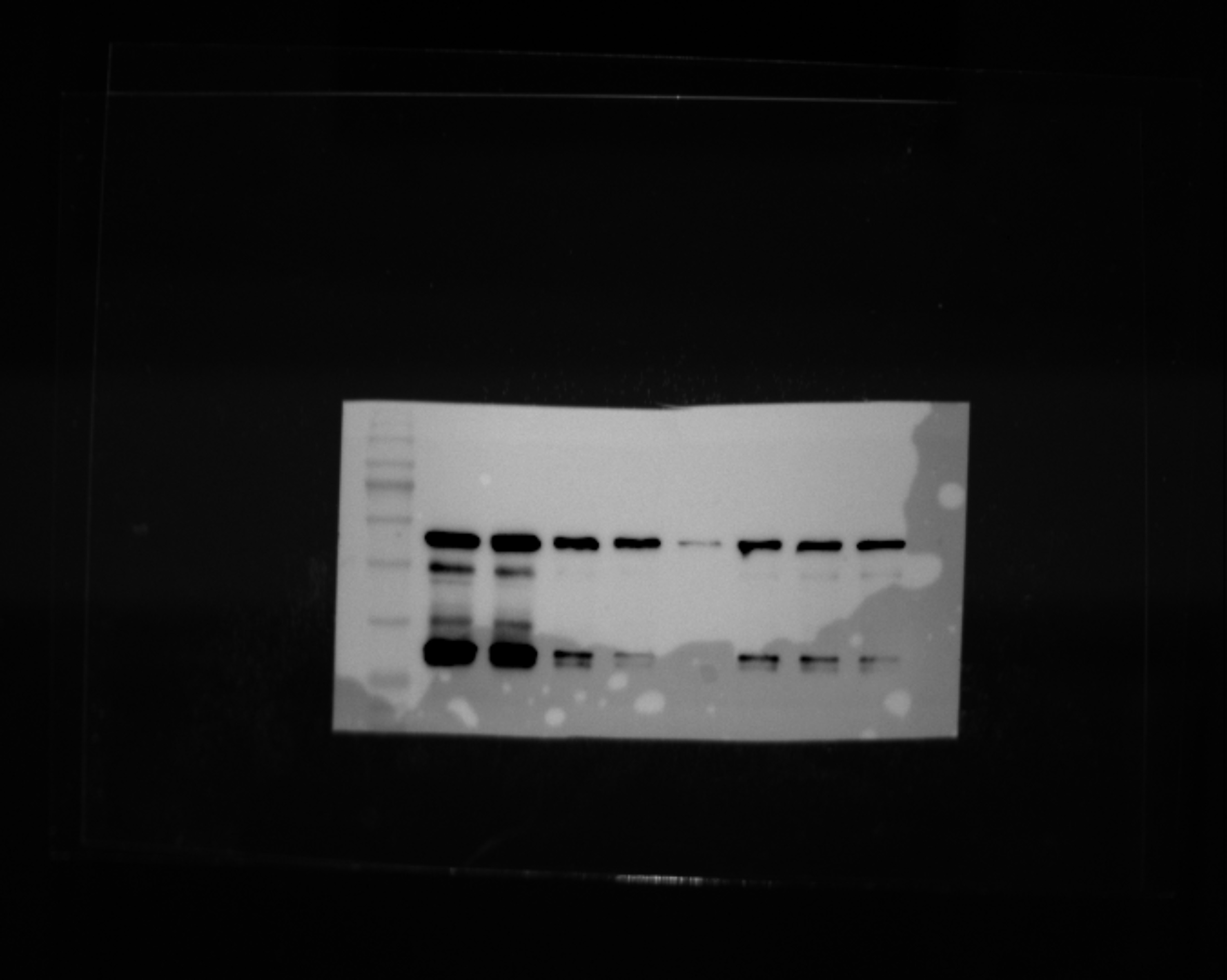

Supplement: Supplementary Material 1. [file jgv-106-02071-s001.zip › Fig 2/Fig 2 D/PEDV_composite.jpg]

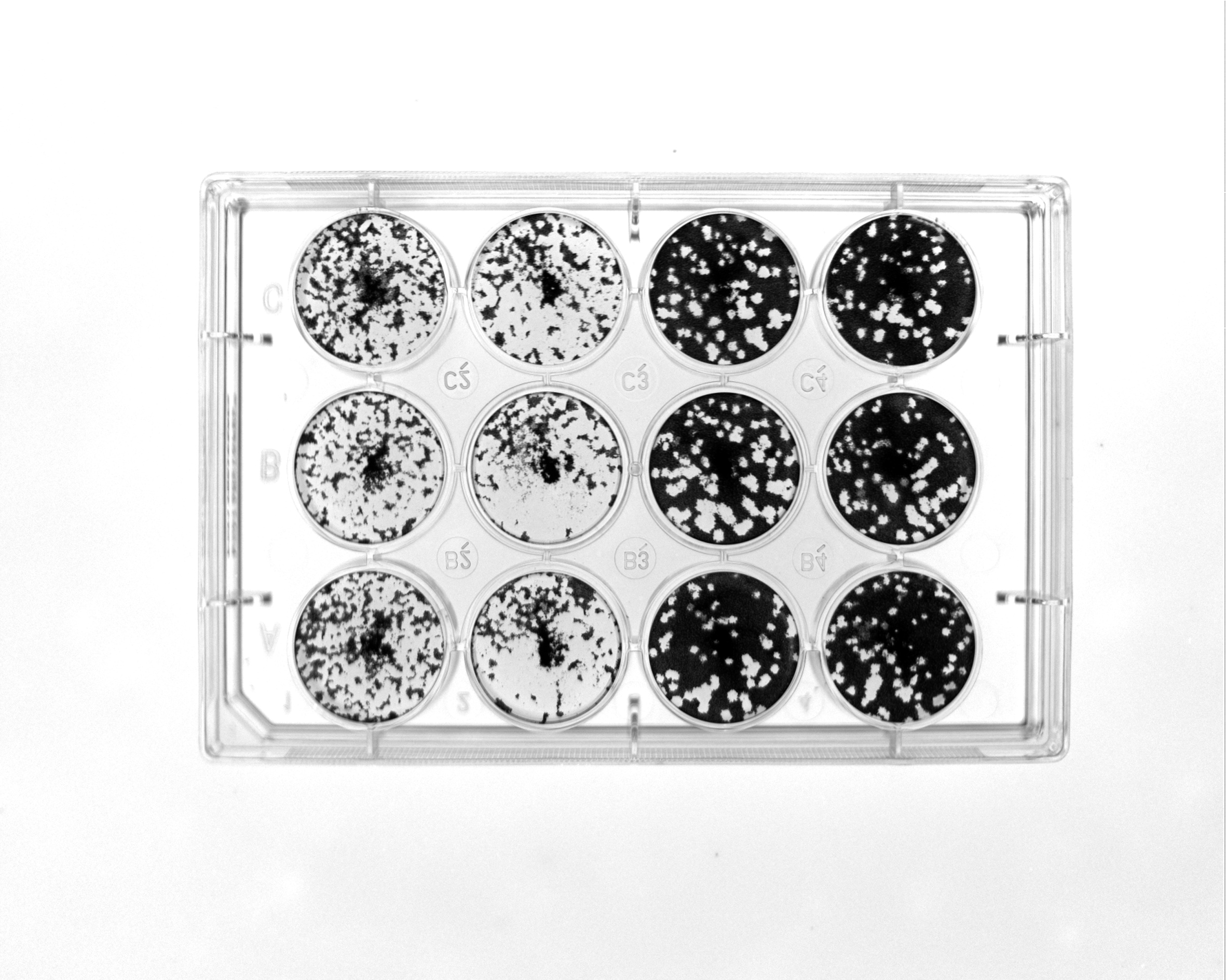

Supplement: Supplementary Material 1. [file jgv-106-02071-s001.zip › Fig 2/Fig 2 E/Non, crRNA1,crRNA2.jpg]

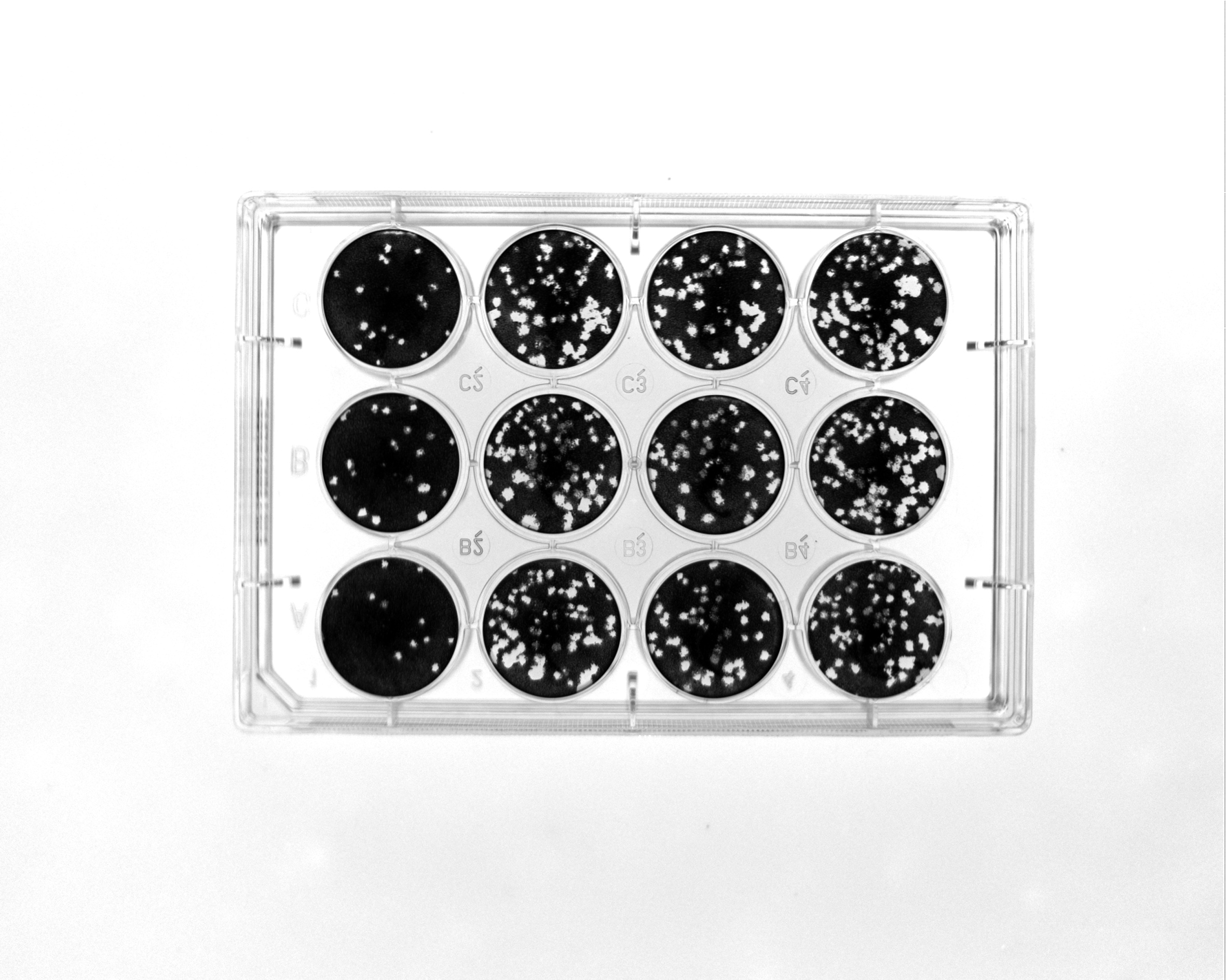

Supplement: Supplementary Material 1. [file jgv-106-02071-s001.zip › Fig 2/Fig 2 E/crRNA3,crRNA4, crRNA5, crRNA6.jpg]

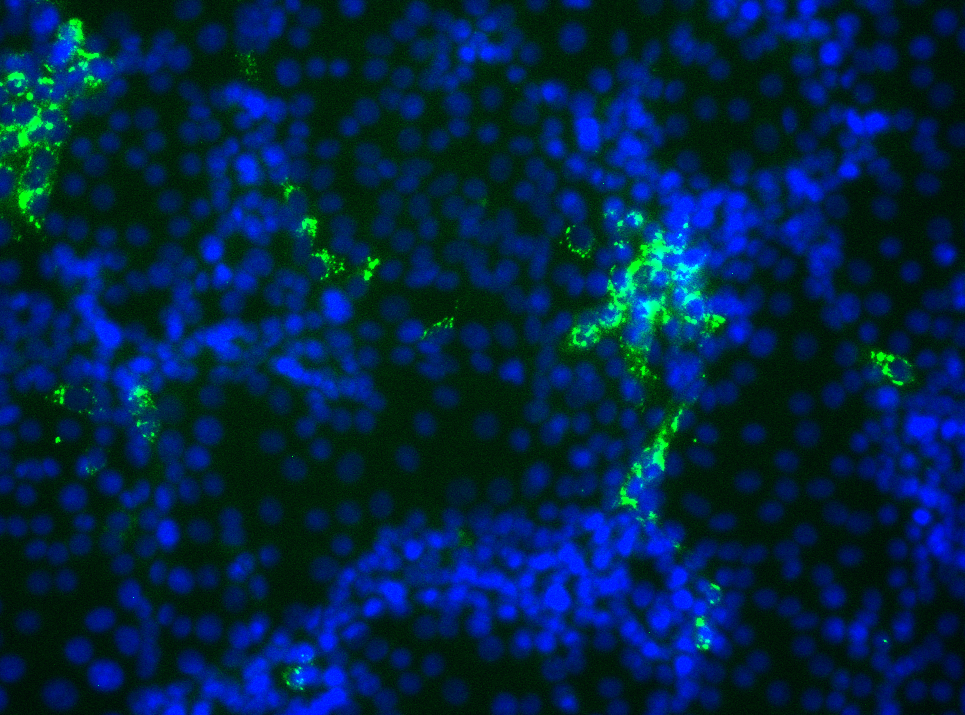

Supplement: Supplementary Material 1. [file jgv-106-02071-s001.zip › Fig 2 B/1_Overlay.tif]

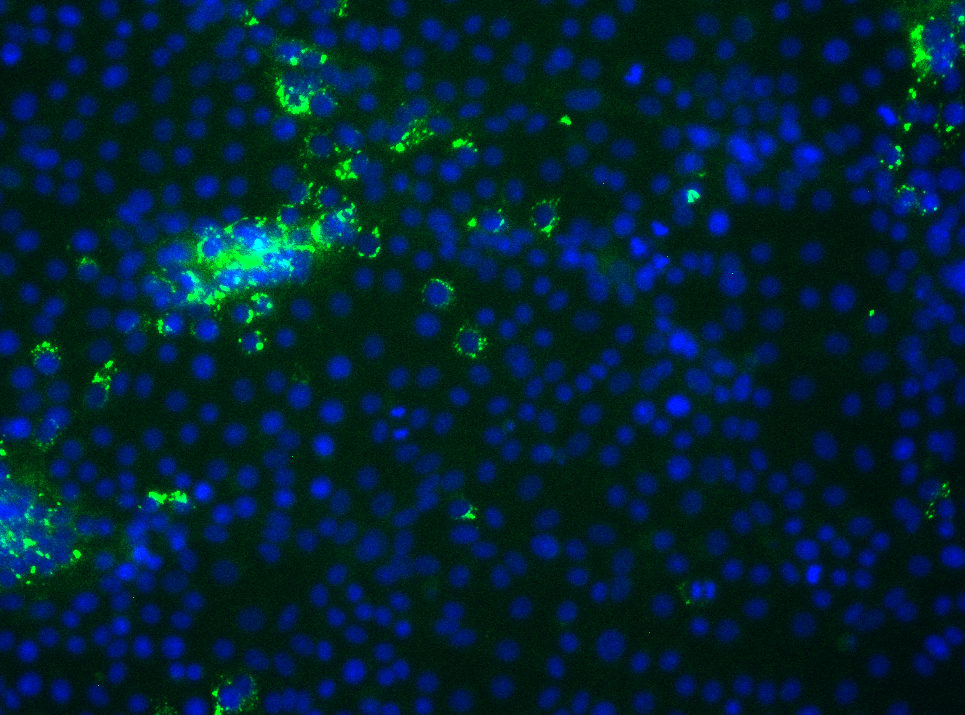

Supplement: Supplementary Material 1. [file jgv-106-02071-s001.zip › Fig 2 B/2_Overlay.tif]

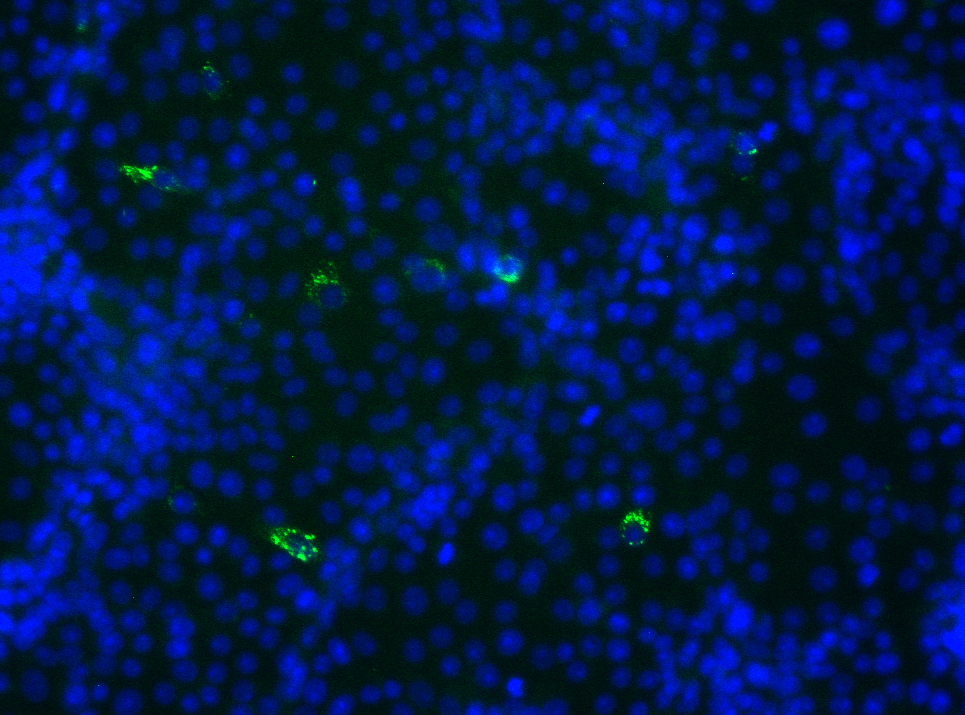

Supplement: Supplementary Material 1. [file jgv-106-02071-s001.zip › Fig 2 B/3_Overlay.tif]

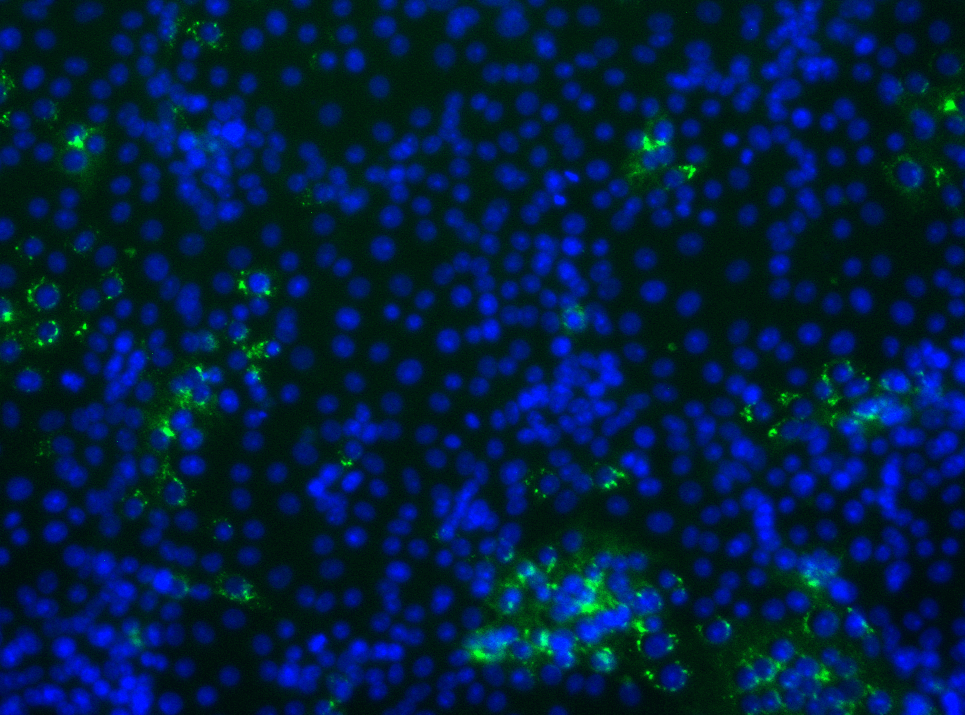

Supplement: Supplementary Material 1. [file jgv-106-02071-s001.zip › Fig 2 B/4_Overlay.tif]

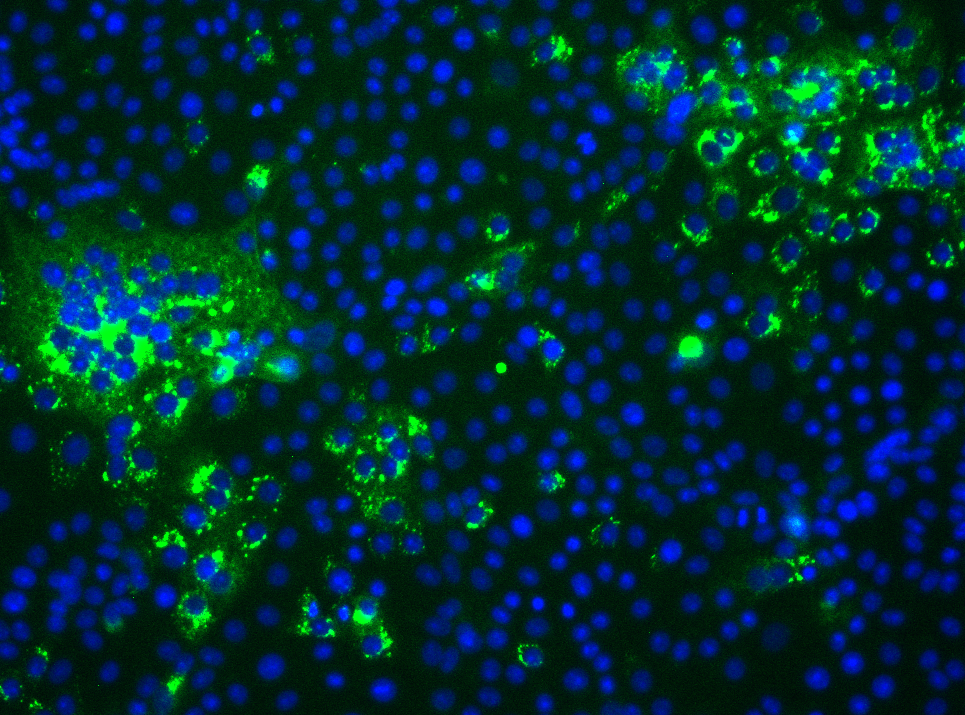

Supplement: Supplementary Material 1. [file jgv-106-02071-s001.zip › Fig 2 B/5_Overlay.tif]

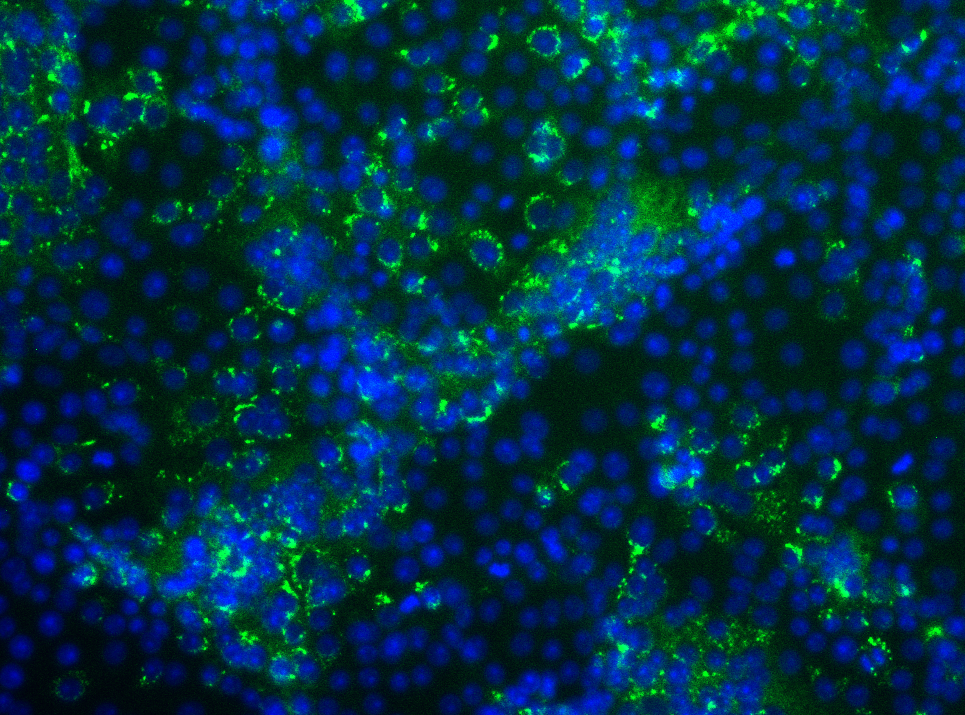

Supplement: Supplementary Material 1. [file jgv-106-02071-s001.zip › Fig 2 B/6_Overlay.tif]

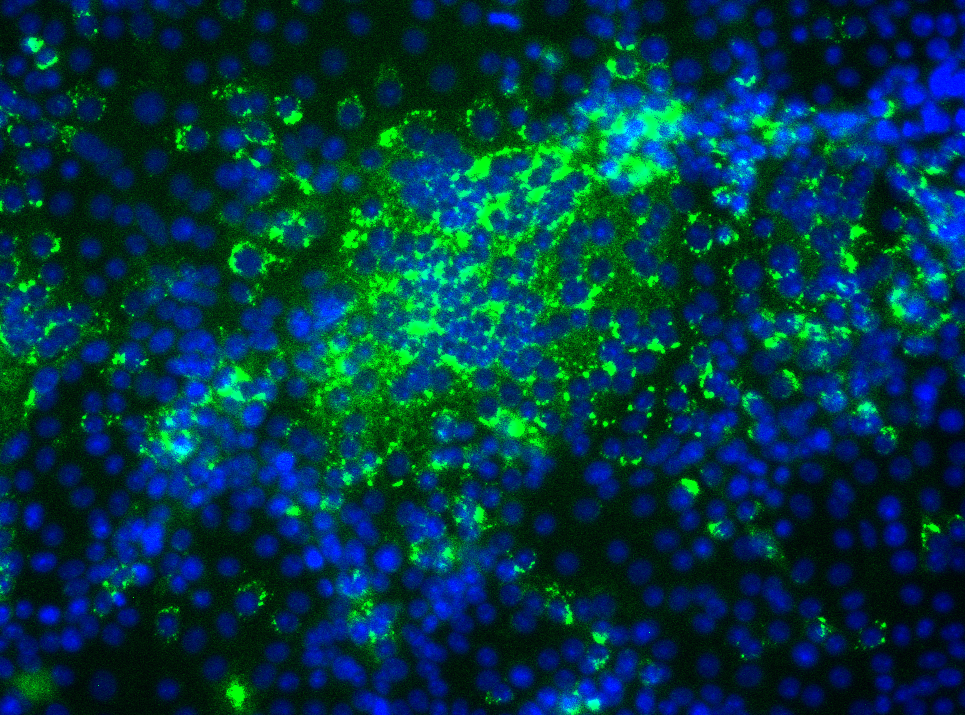

Supplement: Supplementary Material 1. [file jgv-106-02071-s001.zip › Fig 2 B/nt_Overlay.tif]

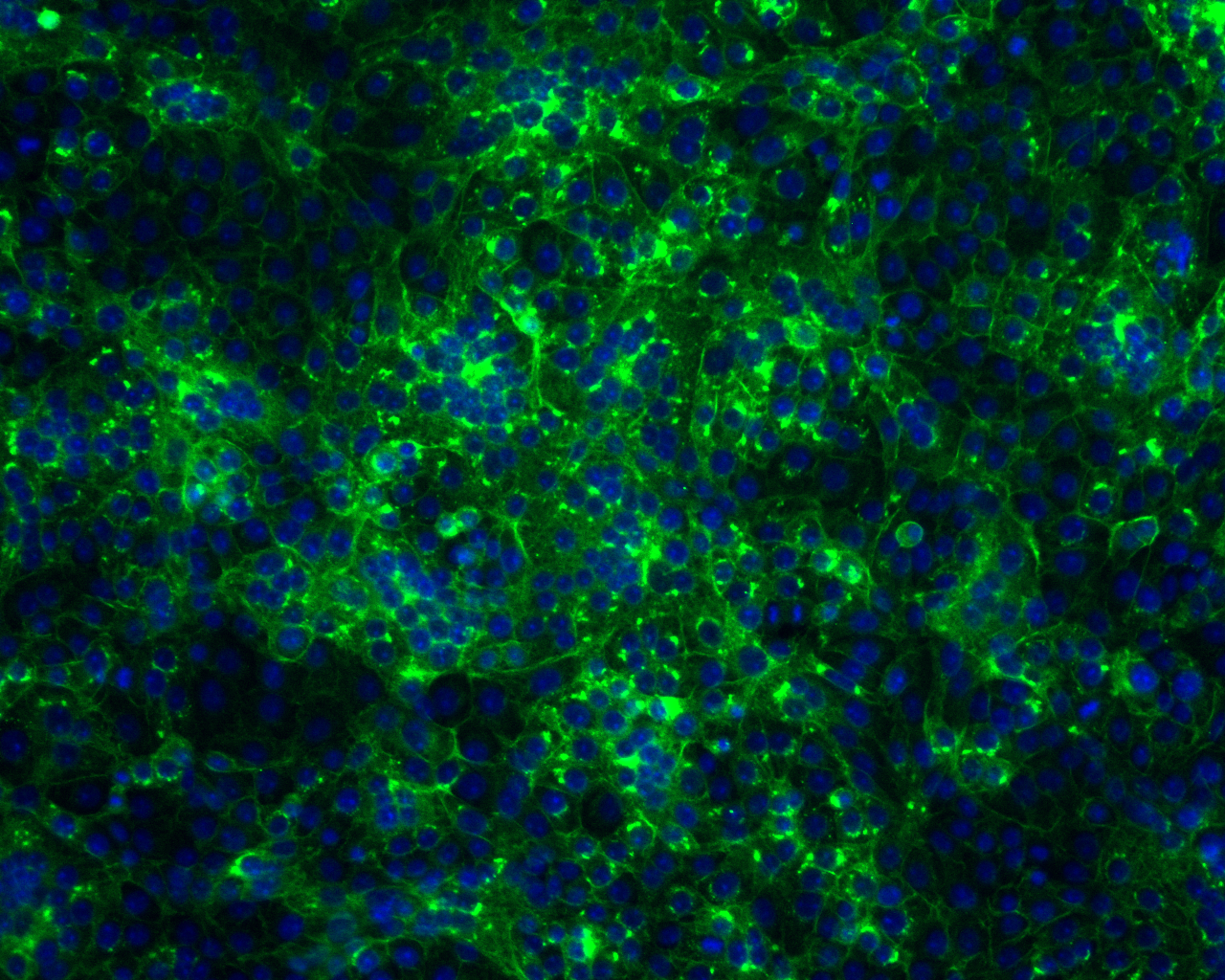

Supplement: Supplementary Material 1. [file jgv-106-02071-s001.zip › Fig 4/Fig 4 B/d-1_Overlay.tif]

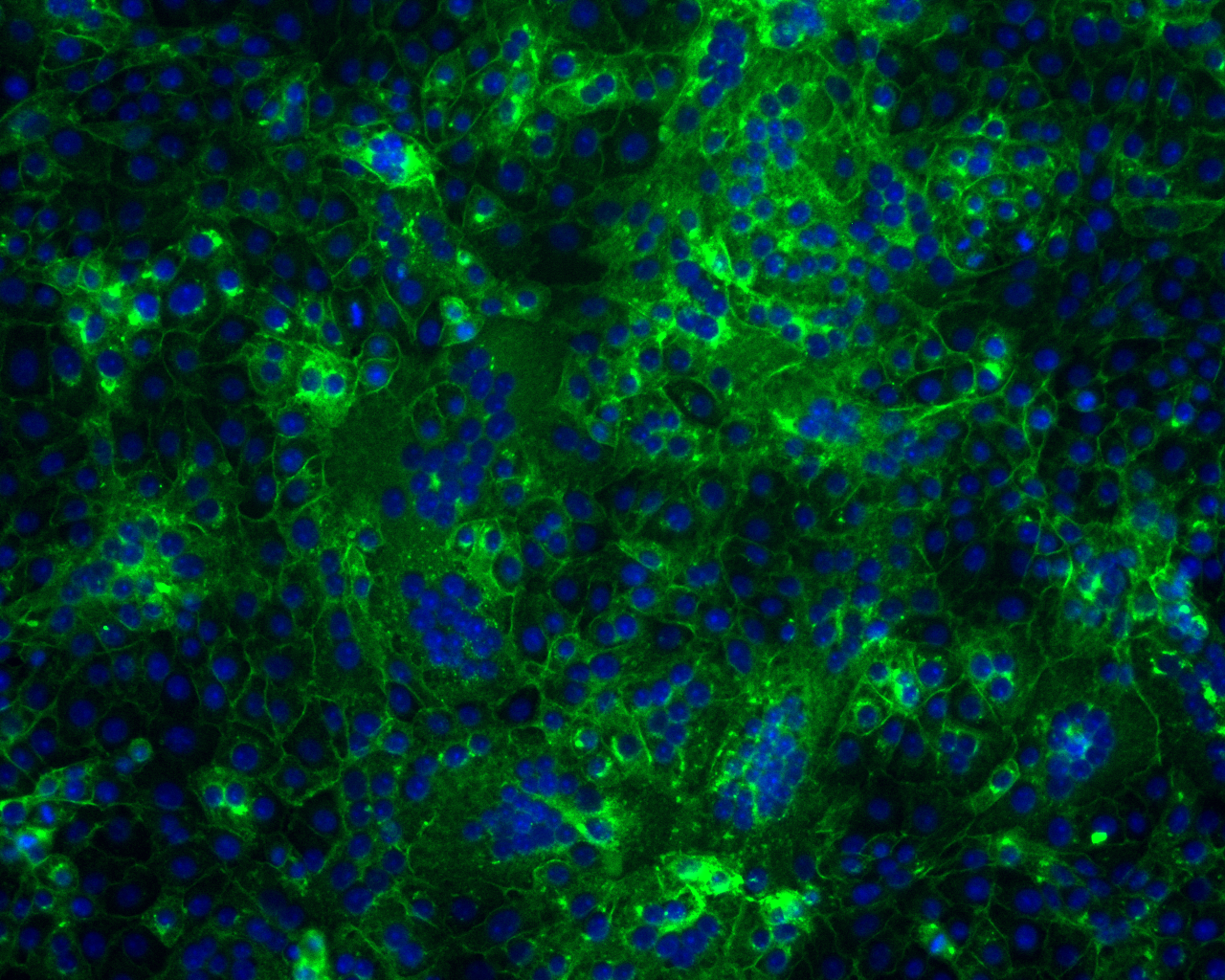

Supplement: Supplementary Material 1. [file jgv-106-02071-s001.zip › Fig 4/Fig 4 B/d2_Overlay.tif]

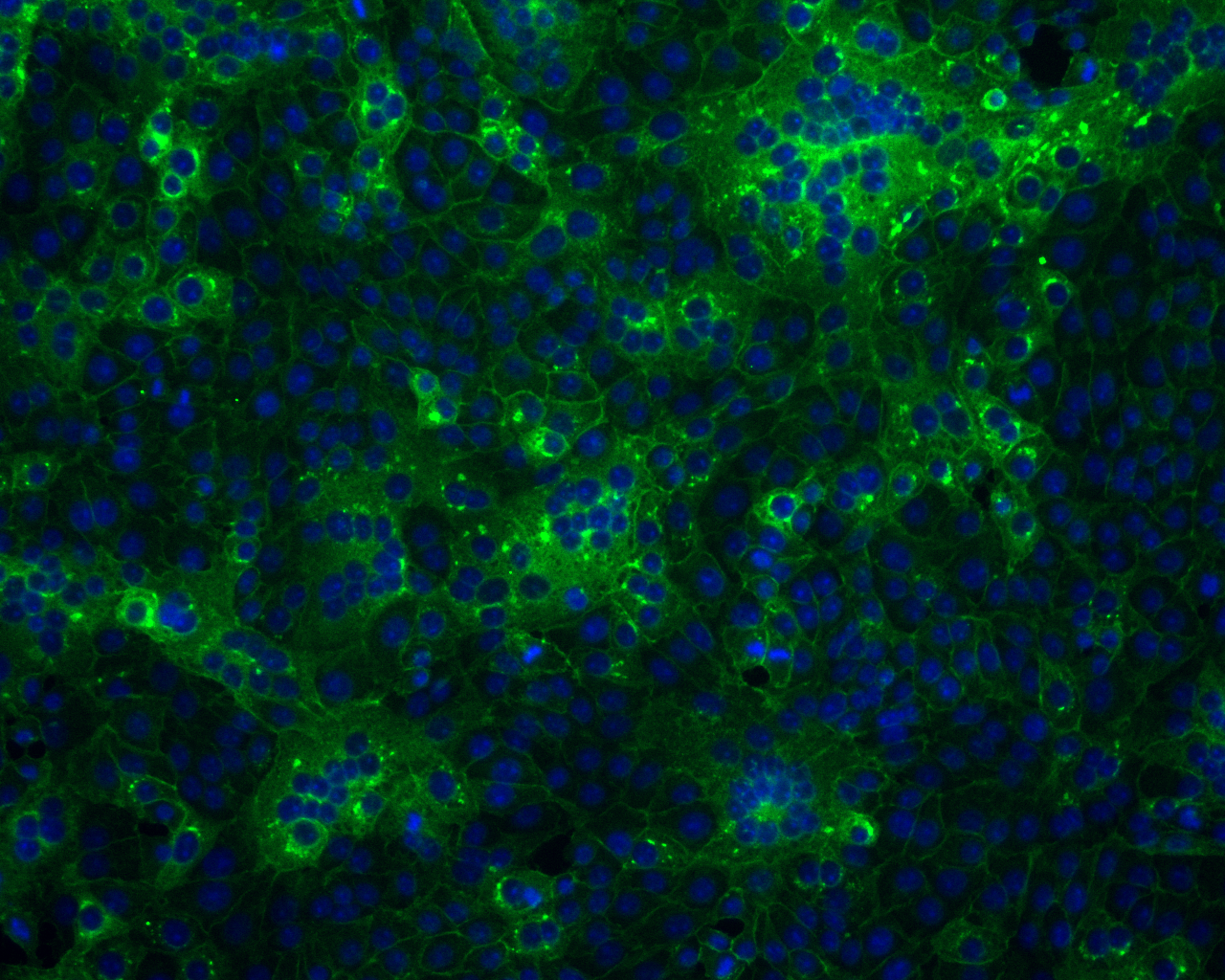

Supplement: Supplementary Material 1. [file jgv-106-02071-s001.zip › Fig 4/Fig 4 B/d3_Overlay.tif]

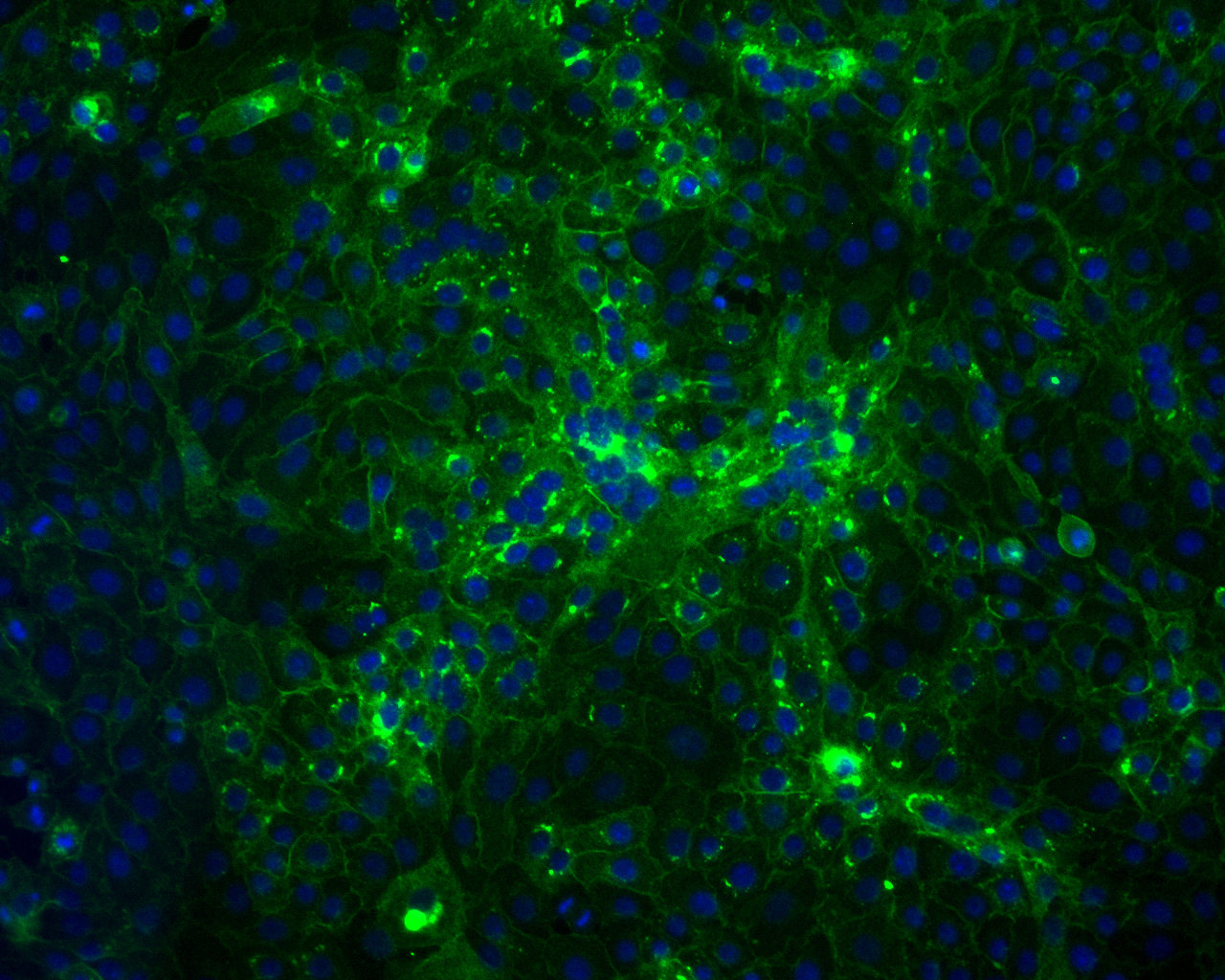

Supplement: Supplementary Material 1. [file jgv-106-02071-s001.zip › Fig 4/Fig 4 B/m1_Overlay.tif]

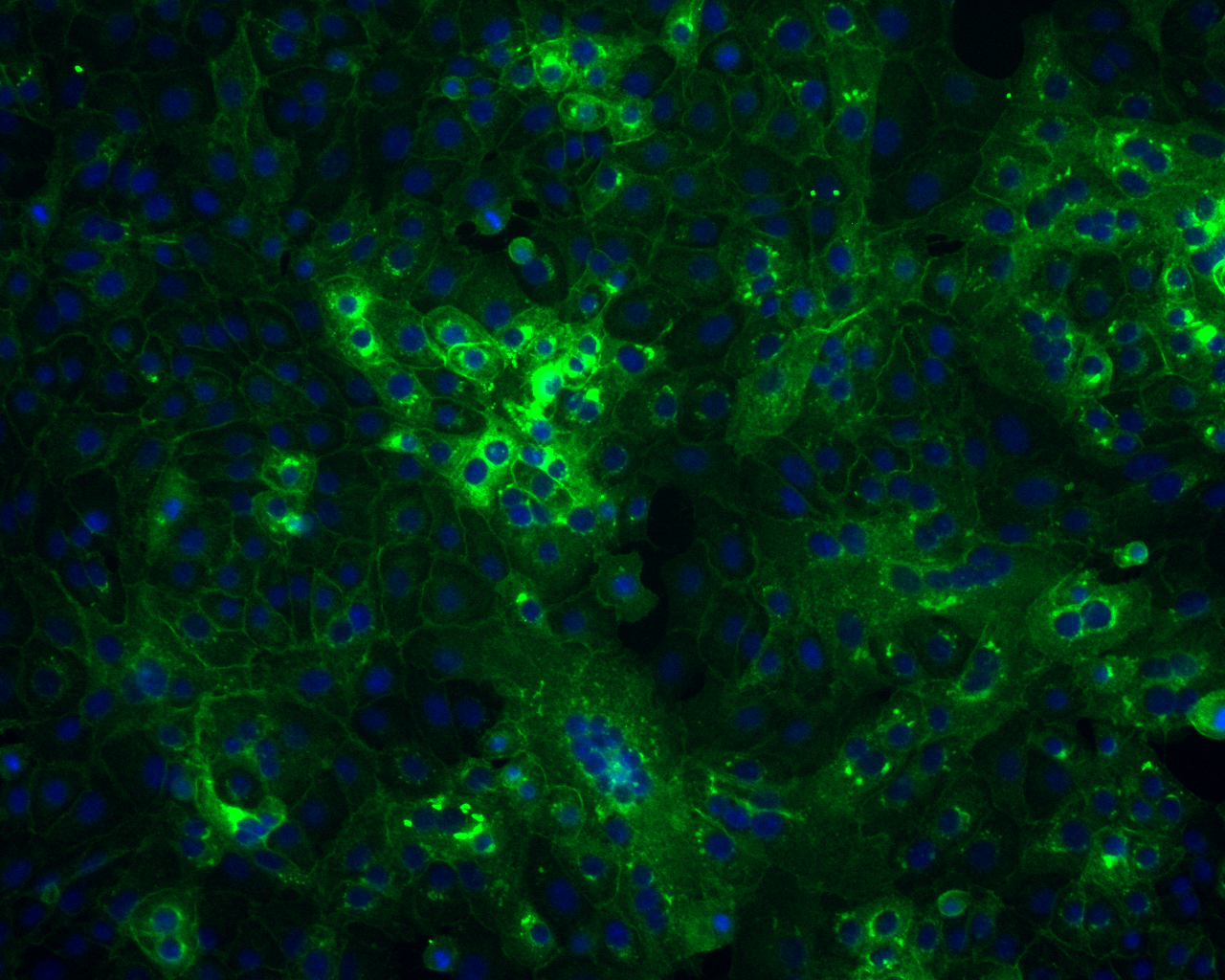

Supplement: Supplementary Material 1. [file jgv-106-02071-s001.zip › Fig 4/Fig 4 B/m2_Overlay.tif]

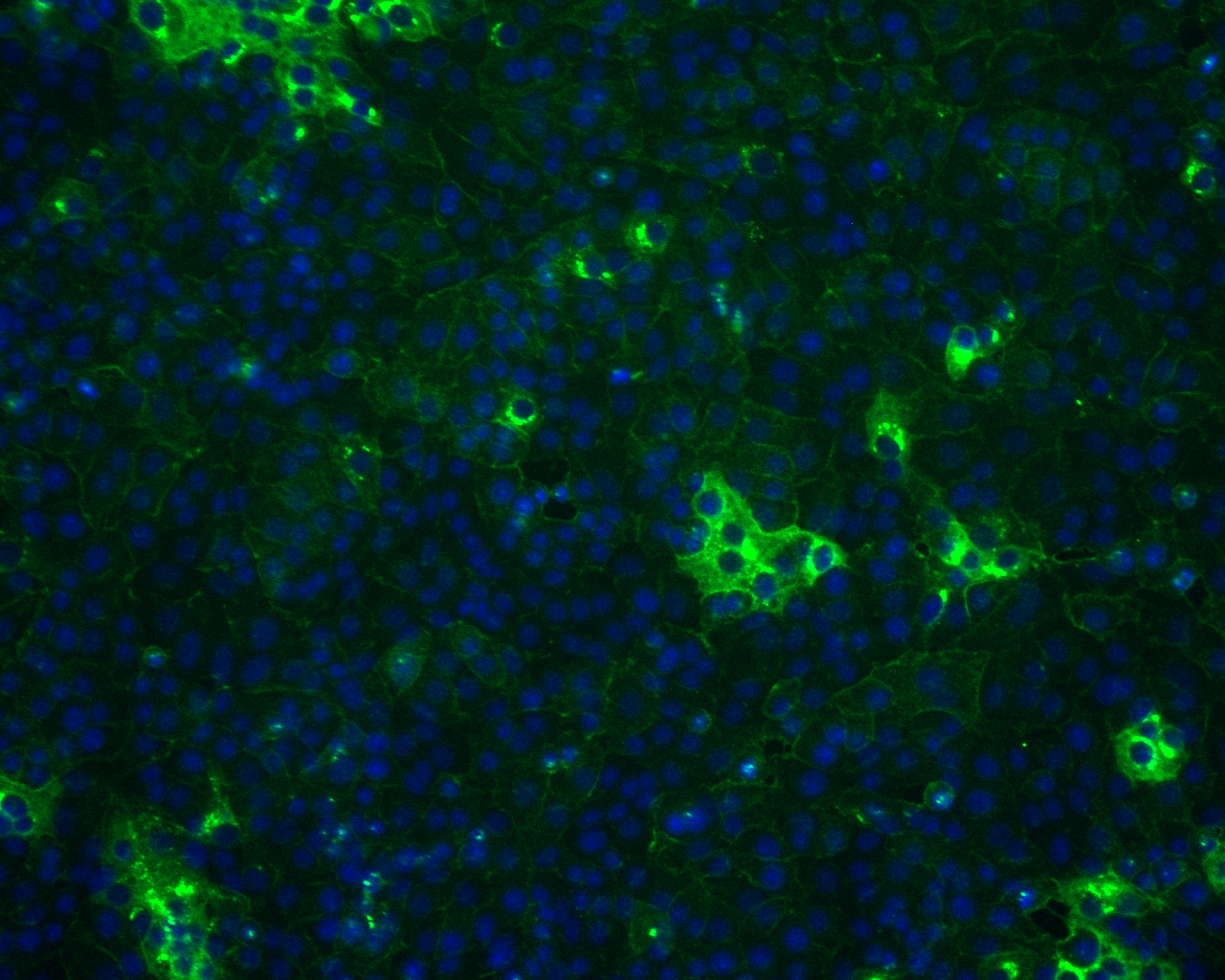

Supplement: Supplementary Material 1. [file jgv-106-02071-s001.zip › Fig 4/Fig 4 B/m3_Overlay(1).tif]
